# Germinal Center Selection and Plasma Cell Differentiation

**DOI:** 10.1111/imr.70169

**Published:** 2026-08-30

**Authors:** Adrien Sprumont, Oliver Bannard

**Affiliations:** ^1^ Medical Research Council (MRC) Translational Immune Discovery Unit, MRC Weatherall Institute of Molecular Medicine John Radcliffe Hospital, University of Oxford Oxford UK

## Abstract

Germinal centers (GCs) are specialized microenvironments in which B cells undergo affinity maturation through iterative cycles of somatic hypermutation, proliferation and selection, generating antibodies with improved antigen‐binding properties. Although GC B cells do not themselves secrete antibody, they differentiate into memory B cells and plasma cells that provide long‐term humoral immunity. GC selection has traditionally been viewed as a process that preferentially expands B cells expressing the highest‐affinity B cell receptors. However, recent studies have revealed that physiological GC responses preserve substantial clonal and affinity diversity, and generate plasma cells spanning a broad range of antibody affinities. Here, we review current understanding of the mechanisms governing GC B cell selection and discuss an alternative framework in which T cell help quantitatively refuels GC B cells rather than acting as a binary gate for cyclic re‐entry. We further explore how antigen organization, antibody feedback and the biophysical context of antigen recognition may reconcile the apparent discrepancy between affinity‐based selection and the maintenance of clonal diversity. Finally, we consider how these concepts may alter our expectations for the plasma cell populations emerging from GCs and discuss their implications for rational vaccine design.

## 1 Introduction

The humoral immune system faces the formidable challenge of mounting protective antibody responses against an almost limitless number of potential pathogens, each presenting multiple antigens and epitopes that may be bound in numerous ways. This diversity problem is addressed through a two‐layered system of clonal development. First, the bone marrow generates via VDJ recombination a large pool of antibody genes to be displayed as B cell receptors (BCRs) by naïve B cells, ensuring that (through chance and with evolutionary pressure having fixed certain biases in germline V, D and J segments and recombination preferences) a fraction of the naïve B cell pool will, at least weakly, recognize any incoming challenge. The second step occurs once the antigenic challenge is defined: this naïve repertoire of antibodies can be fine‐tuned to the eliciting antigen through the process of affinity maturation.

Affinity maturation was first described when Eisen and Siskind noted that anti‐hapten serum antibodies were improving in affinity over the course of an immune response [1]. Affinity maturation takes place within germinal centers (GCs) [2, 3], specialized microanatomical structures that form in reactive secondary lymphoid organs, in which activated B cells undergo iterative rounds of BCR diversification and selection [4, 5, 6]. During this process, somatic mutations are introduced into immunoglobulin genes and variants with improved antigen recognition are preferentially expanded, ultimately generating memory B cells and plasma cells with enhanced protective potential. Although this process is often described as selecting the highest‐affinity BCRs, recent work has revealed a considerably more nuanced picture, particularly in physiological polyclonal responses [7, 8, 9, 10, 11, 12].

Importantly, GC B cells themselves do not directly contribute to humoral immunity. Instead, GCs generate two populations of effector cells: memory B cells and plasma cells [13, 14]. Memory B cells preserve a diversified repertoire for future responses and generally are not selected on the basis of maximal antibody affinity [12, 15]. Plasma cells, by contrast, secrete antibody immediately upon differentiation, and the GC‐to‐plasma cell transition represents an additional step of affinity‐based selection with plasma cells serving as the effectors of humoral immunity [16, 17]. Plasma cells generated from the GC reaction are particularly important as they can secrete antibodies of high affinity which may provide sterilizing immunity to the host. Further, a fraction of the plasma cell population generated in response to a challenge, long‐lived plasma cells (LLPCs), may successfully persist for years, up to the lifetime of the host [18, 19, 20, 21]. Because our vaccines are heavily reliant on humoral protection and because the protection sought is a long‐term one, the efficient generation of GC‐derived LLPCs is critical to the success of vaccination strategies.

Although often studied outside this context, affinity maturation is perhaps better viewed as an optimisation process acting on an entire polyclonal antibody response rather than as a simple attempt to maximize the affinity of individual antibodies, seeking to generate antibodies targeting with useful affinities as many of the foreign antigen's epitopes as is permitted using the pool of weak antibodies provided in the naïve BCR repertoire and the constraints of selection processes [22]. From this perspective, it is perhaps unsurprising that polyclonal responses retain some level of clonal diversity, presumably to avoid the risk of being misdirected and unprotective. The process as a whole relies on GCs determining which BCRs are “best” to select, which they do through competition. This competition may be influenced by a variety of factors including the forms of antigen encountered. As current vaccines rely heavily on the GC reaction and its production of a population of plasma cells that secrete protective antibody, understanding positive selection with these parameters in mind will be critical in optimizing vaccines such that they produce antibodies with the desired properties. Importantly, the optimal antibody response is likely to differ between pathogens. Some vaccine strategies, such as those targeting HIV, require prolonged maturation of particular clonal lineages before plasma cell differentiation [23], whereas others, including malaria vaccines [24], rely on generating exceptionally high titers of neutralizing antibody. HIV vaccines must ultimately also generate high titres of these highly focussed antibodies, but their production may be best deferred until the requisite clonal maturation has occurred.

In this Review, we begin with a discussion of key selection processes that shape the antibody repertoires generated within GCs. We then consider recent advances in our understanding of the plasma cell populations that emerge from GCs, highlighting evidence that these populations are more diverse in both affinity and clonal composition than previously appreciated. To understand this outcome, we will explore how antigen sensing in GCs may cause selection to be influenced by features beyond BCR affinity alone and consider mechanisms promoting the maintenance of antibody affinity and clonal diversity as GC responses progress. Throughout, we present these topics through the lens of our own interpretation of the field, with particular emphasis on areas to which we have contributed or that are particularly relevant to our own research. Rather than proposing a definitive, unified model, our aim is to highlight a perspective of how context may reconcile apparent discrepancies between mechanistic and observational studies, while stimulating discussion and encouraging further investigation into the mechanisms that govern GC output.

## 2 Affinity Maturation Is Underpinned by a Germinal Center B Cell Program

Improvements in antibody affinity are achieved by GC B cells undergoing repeated cycles of immunoglobulin gene somatic hypermutation (SHM), rapid proliferation, and selection of the resulting somatic variants according to the properties of their BCRs [4, 25, 26]. SHM, catalyzed by the enzyme activation‐induced cytidine deaminase (AID), occurs preferentially at hotspot sites within the variable regions of antibody genes; however, it generates amino acid changes without any prior knowledge of their functional consequences for antigen binding, meaning that selection is needed to shepherd survival and expansion of rare somatic variants with improved BCR affinities [27]. This evolutionary process proceeds with remarkable efficiency, with GC B cells often improving their BCR‐antigen affinities (typically measured by the equilibrium dissociation constant, K_D_) by tens or even hundreds‐fold within just 1–2 weeks of responses forming, often through multiple sequential affinity‐enhancing changes [8, 9]. Given this, it is perhaps unsurprising that the process involves tight spatial and temporal coordination of its constituent processes. This coordination is achieved through cyclical transitions between two transcriptionally and functionally distinct cellular states that occupy anatomically segregated compartments, known as dark zones (DZs) and light zones (LZs) [25, 28].

Originally termed centroblasts and centrocytes, the cells of each zone are now commonly known simply as DZ and LZ GC B cells, reflecting the current understanding that they represent dynamic, interconvertible states rather than discrete developmental populations [29, 30, 31]. AID is principally active in periods immediately following mitosis in DZ cells [32], which progress through successive cell divisions with only comparatively short intervening G1 periods [33, 34]. In contrast, most LZ cells are in a relatively quiescent late G1 phase of the cell cycle [30, 35], with small fractions in early S phase, although this distinction is not absolute and some (far less frequent) mitosis events do happen at this stage [30, 36]. Beyond these functionally important behavioral differences, DZ and LZ cells also have distinct phenotypes; DZ cells are commonly identified by high CXCR4 expression [29], and lower expression of various surface markers commonly associated with activation, most notably CD83 and CD86 [31, 37], while LZ cells are characterized by the opposite expression patterns. Beyond being a useful identification marker, high CXCR4 expression by DZ cells is critical for controlling positioning, driving chemoattraction toward an interconnected network of CXCL12‐expressing Reticular Cells (CRCs, also known as DZ follicular dendritic cells, FDCs) present in DZs [38, 39, 40]. Downregulation of CXCR4 by LZ cells readjusts chemokine responsiveness such as to favor CXCR5‐mediated attraction to the chemokine CXCL13 expressed by LZ FDCs [29]. These FDCs also play a critical role in affinity maturation by retaining immune complexes composed of antigen (often intact) decorated by antibody and/or complement components, which they achieve due to very high expression of complement (CR1/2, or CD21/35 by CD nomenclature) and Fc (FcgRIIB, or CD32B) receptors [28, 41]. To avoid confusion, let us note that FDCs (LZ and DZ) are stromal cell components of the lymphoid tissue rather than hematopoietic‐derived immune cells [42].

We observed some years ago that GC B cells undertake the usually DZ‐associated processes of proliferation, SHM, and CD83/CD86 downregulation even when they are trapped in LZs through CXCR4 deficiency, identifying that many of the DZ and LZ cell defining behaviors are regulated by intrinsic cellular programs rather than zonally‐derived cues [38]. Moreover, frequencies of CXCR4‐deficient DZ‐ and LZ‐like cells were close to normal, prompting us to propose that the DZ cell program incorporates an intrinsic cellular timer, or perhaps more accurately, a division counter [5], that determines when they should transition back to the other state [4]. A body of work has since established that this programmed behavior provides a central framework for affinity maturation because it allows the strength of affinity‐based selection to be translated into proportional differences in subsequent proliferation and SHM [33, 43, 44, 45], thereby shaping the evolutionary trajectories of selected B cell clones, a concept to which we return later. In addition, the DZ and LZ programs also coordinate the extensive molecular remodeling required for cells to efficiently undertake their respective roles, switching between proliferative and antigen‐presenting states [31, 37]. For example, the transition from the LZ to the DZ is accompanied by increased expression of the E3 ubiquitin ligase March1, which, analogous to its role in immature dendritic cells [46, 47, 48], promotes accelerated turnover of MHC Class II and CD86 when antigen presentation is no longer required [22]. Conversely, upon returning to the LZ, GC B cells downregulate March1 while upregulating its antagonist CD83 and increasing de novo MHCII synthesis, thereby restoring stable surface expression of antigen‐presenting machinery for interaction with cognate CD4 T cells. Whether regulation of other ubiquitin ligases and/or post‐translational modifiers occurs in analogous ways to support highly dynamic GC B cell behavior is not currently known but seems likely.

The existence of an intrinsic GC program naturally raises the question of how it is regulated on a molecular level. Although the DZ program behaves as a coordinated cellular state, its execution depends on multiple regulatory pathways operating in parallel. The Forkhead box protein O1 (FoxO1) transcription factor coordinates much of the DZ transcriptional program, as GC B cells lacking FoxO1 adopt a predominantly LZ‐like phenotype (e.g., CXCR4^low^ CD83^high^ CD86^high^) [49, 50, 51]. This association with high rates of proliferation is highly unusual for FoxO1, because its canonical role outside of the GC is in promoting states of quiescence, perhaps reflecting its unique collaboration with Bcl6, with which it shares many promoter and enhancer binding sites [49]. FoxO1 activity is subsequently antagonized in LZ cells through PI3K–AKT‐mediated phosphorylation and nuclear exclusion. Nevertheless, FoxO1 is not an all‐encompassing regulator of DZ biology. AID expression and activity, as well as the cell‐cycle progression normally associated with the DZ, remain largely intact in phenotypically LZ‐like GC B cells that are FoxO1‐deficient or that have hyperactive PI3K signaling [49, 50, 51]. This uncoupling of phenotype and proliferative behaviors is particularly striking because CXCR4 downregulation (defining the DZ to LZ transition) normally occurs during a defined late‐G1 stage of the cell cycle [34, 35]. How such tight coordination of different pathways occurs is not currently known, although we will touch on aspects of it in subsequent sections.

## 3 Germinal Center Selection

The selection of GC B cells following SHM has traditionally been viewed through the lens of a single LZ‐associated selection step, in which B cells compete on the basis of BCR affinity for cyclic re‐entry, the process by which positively selected LZ cells re‐enter S phase and return to the DZ for further rounds of proliferation and somatic hypermutation [4, 25, 52]. In this prevailing model, DZ cells exit the cell cycle and enter a state of quiescence several hours before returning to the LZ, where they compete for limiting signals that promote survival and drive cyclic re‐entry; in the absence of sufficiently strong selection, their default fate is apoptosis. Because these signals are considered limiting, B cells that acquire more antigen are predicted to secure a disproportionate share of the cues required for survival and cell‐cycle re‐entry, thereby biasing selection toward higher‐affinity clones and somatic variants within clones. According to this framework, only cells that surpass the selection threshold re‐enter S phase and return to the DZ, thereby completing cyclic re‐entry and initiating another round of proliferation and somatic hypermutation [53]. Apoptotic GC B cells are rapidly cleared by tingible body macrophages, a specialized efferocytic tissue resident cell [54, 55].

**FIGURE 1:**
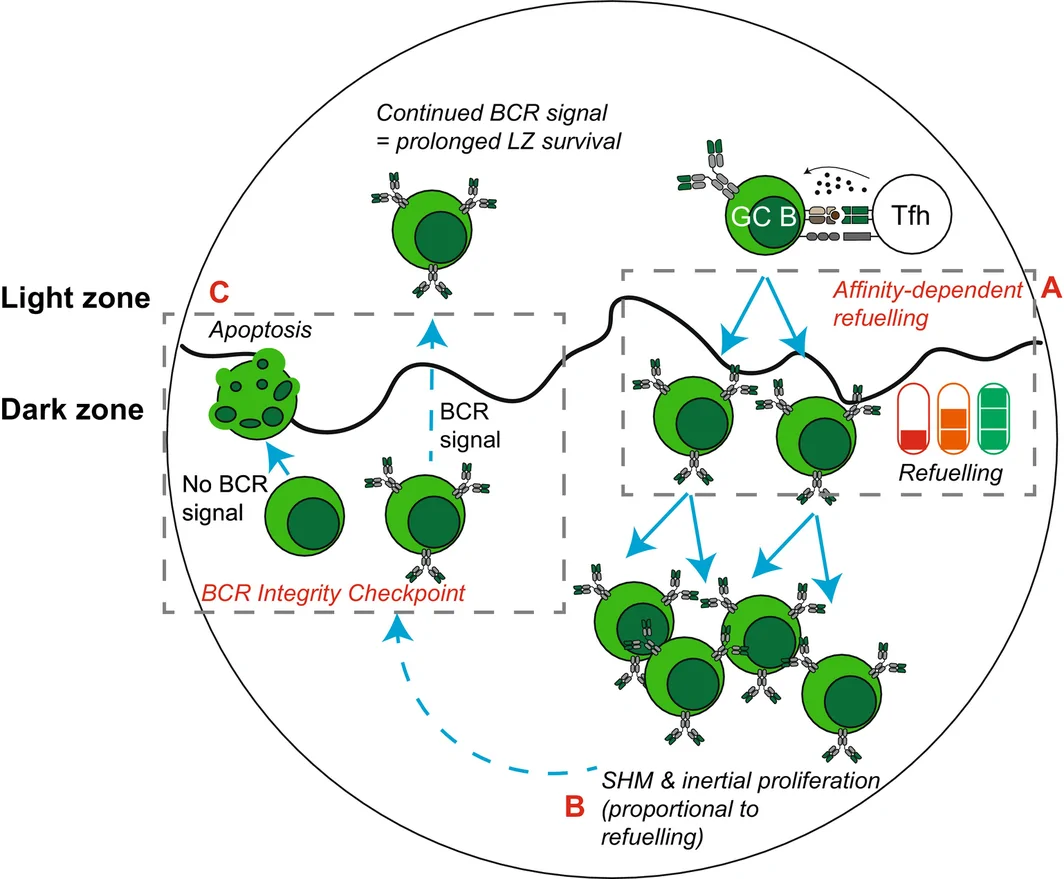
Two‐layered selection in germinal centers. (A) LZ GC B cells extract immobilized antigen tethered to FDC membranes for presentation to Tfh cells. The cumulative signal acquired through multiple short interactions with Tfh cells refuels LZ GC B cells. The receipt of help is limited through competition from other B cells and by intrinsic BCR avidity coupled with antigen availability. Cyclic re‐entry is initiated independently of T cell help, allowing refueling to occur across a broad dynamic range. LZ B cells presenting greater amounts of peptide–MHCII receive stronger refueling. (B) Refueling received in LZs programs and facilitates proliferation subsequently completed in DZs, such that expansion occurs in accord with BCR affinity. DZ cell division is typically accompanied by SHM; however, GC B cells undergoing extensive clonal bursts transiently silence AID activity, preserving the selected BCR sequence during its expansion. (C) DZ cells that acquire deleterious antibody gene mutations downregulate surface BCR expression and undergo apoptosis before returning to the LZ. Once back in the LZ, surviving GC B cells re‐enter this cycle. LZ BCR signaling promotes LZ cell survival.

Our understanding of the limiting factor over which GC B cells compete has evolved substantially over time. Early models placed particular emphasis on direct competition for antigen displayed on FDCs [25], with cognate linked T‐cell help subsequently providing an essential permissive signal for continued survival and differentiation [56]. However, live imaging studies demonstrated that GC B cells show little evidence of pausing on FDCs in such ways that are likely to physically exclude others from antigen‐bearing surfaces, as would be required for competition [30]. The same studies also revealed that interaction between GC B cells and Tfh occurs quite infrequently in LZs and tends to be transient in nature, raising the possibility that access to T cell help, rather than antigen itself, constitutes the principal competitive bottleneck. This was consistent with earlier in vitro studies using engineered B cell lines that established that antigen capture and presentation to helper T cells scale with BCR affinity, providing a mechanistic link between antigen binding and differential access to T‐cell‐derived selection signals [57]. A landmark study by Victora and colleagues subsequently provided direct experimental proof that selectively augmenting peptide–MHCII presentation by GC B cells is sufficient to confer a marked competitive advantage over neighboring GC B cells, demonstrating that differential antigen presentation alone is sufficient to shape GC selection [31]. It is thought that LZ GC B cells presenting more peptide antigen will distract T cells away from other B cells of lower BCR affinity and thereby disadvantage them. We refer readers to several excellent reviews that discuss these studies in greater detail [5, 58, 59]; here, we provide only the background necessary to frame the below discussions, where we consider recent findings indicating that GC B cell selection is not confined to a single LZ‐based process and outline an alternative framework to the cyclic re‐entry model for understanding affinity‐dependent positive selection (Figure 1).

### 3.1 Testing B Cell Receptor Integrity Following Somatic Hypermutation

We have recently learned that the testing by GC B cells of newly mutated BCRs following SHM comprises at least two discrete selection steps: the first, where BCR integrity is assessed, operates before or during transition from the DZ to the LZ [34, 60], and facilitates the task of the later step of affinity‐based selection in the LZ. GC B cells accumulate somatic mutations at an estimated rate of approximately one mutation per 1000 bp per cell division, meaning that, across the paired immunoglobulin heavy‐ and light‐chain variable regions, each daughter cell has a roughly two‐thirds probability of acquiring at least one mutation following mitosis. However, only a small minority of these mutations are expected to improve antigen‐binding affinity (~1%–2%), whereas substantially larger fractions either reduce affinity (~15%) or compromise BCR expression or stability through deleterious coding changes (~30%) [61, 62]. Consequently, an important function of GC selection is the efficient elimination of the large numbers of non‐functional or disadvantageous variants generated during SHM—which we now know they achieve before LZ entry.

Using Ighv sequencing of GC B cells that express a pre‐arranged “SW_HEL_” hen egg lysozyme‐specific BCR (Igh knock‐in/Igk transgenic mice), we mapped the phenotypic distribution of cells harboring SHM‐induced premature stop codons that abolish BCR expression [34]. Premature stop codons were substantially more frequent in DZs than in LZs, rather than accumulating in the LZ as would be expected if non‐selected GC B cells primarily died following failure to complete cyclic re‐entry. Moreover, stop codon‐containing cells within the DZ had frequently lost most or all surface BCR expression, and BCR‐low or negative DZ cells were highly enriched among apoptotic cells, as identified by active caspase‐3 staining. Independently, Mayer et al. developed a FRET‐based apoptosis reporter to map the spatial distribution of GC B cell death, similarly identifying substantial apoptosis within DZs and demonstrating that cells that lose BCR function through SHM are predominantly eliminated within this compartment [60]. Collectively, these findings support a hierarchically organized model of GC selection, in which an initial quality‐control checkpoint operating within the DZ removes cells expressing non‐functional or severely impaired BCRs before the canonical LZ affinity‐selection program. Moreover, because GC B cells typically complete an entire LZ–DZ cycle in approximately 24 h [33], newly generated receptors must be expressed on the cell surface rapidly enough for their functionality to be tested before cells re‐enter the LZ. Whether this is achieved through gradual dilution of pre‐existing receptors during cell division or through an active, transient BCR turnover mechanism remains unknown.

The molecular basis of this negative “quality control” selection remains uncertain. BCR signaling is substantially rewired in GC B cells compared with other B cell populations, with elevated phosphatase activity attenuating canonical signaling while shifting downstream responses toward PI3K‐Akt‐dependent pathways [63, 64]. Furthermore, the DZ contains relatively little immune‐complex‐bearing antigen [28]. One possibility therefore is that receptor expression is monitored through an antigen‐independent tonic signaling mechanism analogous to that supporting naïve B cell survival [65, 66, 67]. However, this explanation is difficult to reconcile with the markedly different kinetics. Whereas naïve B cells survive for several days after BCR loss, BCR‐deficient GC B cells undergo apoptosis within only a few hours (if not less). Moreover, DZ GC B cells are relatively resistant to pharmacological inhibition of Btk and genetic loss of Syk, despite their striking sensitivity to complete loss of BCR expression [35, 63, 68].

A possible explanation is that apoptosis reflects a distinct pre‐LZ BCR‐dependent checkpoint rather than a general requirement for BCR expression throughout the DZ. Consistent with this possibility, DZ cells preferentially undergo apoptosis during late G1, several hours after mitosis, corresponding approximately to the period when BCR expression is at its lowest (presumably among cells not returning to LZs) and to when cells begin downregulating CXCR4 as they prepare to return to the LZ [34]. However, this alone does not explain how loss of BCR expression is translated into apoptosis if DZ cells are not generally dependent on Btk or Syk activity [35, 63, 68]. This could be reconciled if GC B cells begin reacquiring aspects of the LZ signaling state before fully relinquishing their DZ phenotype, creating a transitional state—possibly initiated immediately following FoxO1 phosphorylation—in which cells become progressively dependent on BCR‐mediated PI3K‐Akt signaling for survival. A related, but mechanistically distinct, possibility is that DZ cells transiently require BCR signaling upstream of FoxO1 inactivation, with this serving as a dedicated quality‐control checkpoint. In this model, successful BCR expression permits PI3K‐Akt activation, leading to FoxO1 phosphorylation and inactivation and thereby licensing progression toward the LZ state, whereas cells that fail to express a functional BCR are unable to initiate this signaling event and instead undergo apoptosis.

Both these possibilities are consistent with the observed temporal association between BCR loss, late‐G1 apoptosis and initiation of LZ migration, and each provides a plausible explanation for how receptor integrity could be coupled to GC zone transitions. Nevertheless, neither model fully explains how loss of BCR expression is molecularly sensed or how this information is translated into activation of the apoptotic machinery. Future studies should therefore determine whether this checkpoint depends on tonic BCR signaling, transient reacquisition of antigen responsiveness, or an entirely distinct mechanism linking receptor expression to the molecular program governing DZ exit. We also cannot exclude at this stage involvement of some form of non‐canonical tonic BCR signaling or that sensing of BCR loss is indirect, for example due to protein quality control mechanisms in the endoplasmic reticulum [69, 70].

Once GC B cells expressing non‐functional receptors have been purged, the remaining population must be discriminated on the basis of differences in BCR affinity, which occurs in LZs.

### 3.2 Light Zone Selection Through Affinity‐Dependent Refueling

Our own work revisited a central tenet of GC selection; that competing LZ GC B cells must receive a threshold level of T cell help to pass a selection bottleneck and initiate cyclic re‐entry, implying a fundamentally binary outcome (cyclic re‐entry or not) [35]. A key prediction of such models is that LZ cells that compete poorly for cognate T cell interactions, owing to inferior peptide–MHCII presentation, will stall in a quiescent G1 phase and ultimately undergo apoptosis without progressing back into cell cycle. This question was tackled using mice carrying the Fucci2 fluorescence‐based cell cycle reporter gene [71], which permits unambiguous identification of cells initiating S phase from the late‐G1 state characteristic of LZ cells. These mice were bred to also carry genes that permit tamoxifen‐induced deletion of MHCII, thereby allowing antigen presentation to be temporally and selectively halted in cohorts of B cells within established GCs. Experiments were performed using mixed bone‐marrow chimeras that also contained wildtype cells from mice with only the Fucci2 alleles, such that MHCII‐deleted cells competed within GCs where neighboring B cells presented peptide antigen at normal levels. Unexpectedly, LZ GC B cells continued to pass the G1‐S checkpoint that defines the initiation of cyclic re‐entry even after MHCII deletion, which would prevent them establishing cognate Tfh cell interactions. In contrast, these same MHCII‐deleted LZ cells failed to upregulate expression of the transcription factor BATF, and also did not measurably express c‐Myc when BCR signaling was pharmacologically inhibited (using Ibrutinib, a Btk inhibitor) alongside T cell engagement.

These findings challenge the central concept that cyclic re‐entry is obligatorily gated by receipt of Tfh help, while confirming that cognate T cell help is required to induce key transcriptional programs critical to normal downstream DZ function. Collectively, this suggests that the key role of Tfh cells, rather than being gate keepers of cyclic re‐entry, is to “refuel” GC B cells in preparation for further expansion by inducing the metabolic and biosynthetic programs that support subsequent proliferation. An important consequence of uncoupling cyclic re‐entry from T cell‐mediated refueling is that GC B cells no longer face a threshold requirement for S‐phase entry. Instead, T cell help can operate across a broad dynamic range proportional to peptide‐MHCII abundance, with varying amounts of T cell help being continuously integrated and translated into quantitative differences in the magnitude of subsequent DZ proliferation [33, 43]. Consistent with this, even LZ cells with comparatively low BCR affinity express c‐Myc, supporting that T‐cell help is interpreted as a graded quantitative input rather than an all‐or‐none license for proliferation [36].

Mechanistic studies have defined several pathways through which quantitative instruction is encoded. In particular, the number and duration of T‐cell contacts determine the amount of c‐Myc activity in GC B cells by modulating the frequency of *Myc* transcriptional bursts [72, 73, 74, 75], while also proportionally regulating mTORC1 activation [76]. These pathways are transiently induced in LZ cells during selection but are largely extinguished before, or very soon after, DZ re‐entry. Consequently, they are not thought to directly regulate cell‐cycle progression during the ensuing proliferative phase but rather precondition GC B cells by establishing the metabolic, biosynthetic and translational programs that determine the magnitude of subsequent DZ expansion. The execution of this proliferative program relies on DZ‐specific cell‐cycle machinery, which involves maintaining expression of cyclin D3 to complex with CDK4/6 and drive sequential G1‐S transitions without need for further mitogenic stimulation, a phenomenon termed inertial growth [77, 78]. This adaptation makes GC B cells uniquely suited to refueling‐dependent programmed expansion. Although the mechanisms controlling the abundance and persistence of cyclin D3 remain incompletely understood, they may reflect translational or post‐translational programs established at the time T‐cell help is received.

Accordingly, the level of T cell‐mediated refueling LZ cells receive is expected to determine the magnitude of subsequent DZ expansion. Consistent with this, GC B cells complete an average of two cell divisions during each DZ phase [33, 58], while exceptionally strong selection events initiate much larger “clonal bursts” that may comprise up to an estimated 10 divisions [44]. It was recently discovered that AID activity is transiently suppressed during some larger bursts, restricting SHM for most of the proliferative program—with reactivation only (or preferentially) occurring during a DZ cell's final G1 phase [44, 45]. This temporal separation minimizes the accumulation of deleterious mutations during extensive clonal expansion, permitting diversification to resume only once cells carrying the selected antibody gene variant have gained their proliferative advantage. Whether this phenomenon is unique to large clonal bursts or also occurs in a more graded manner during smaller DZ expansions remains unknown [8]. This adds preferential sequence preservation to survival and expansion as a central selection process through which affinity maturation is achieved.

We must acknowledge for more casual readers that one important aspect of this refueling model, that cyclic re‐entry may be initiated in the absence of cognate T‐cell‐dependent selection cues, is not yet widely accepted. This is not surprising given that the conclusion currently rests largely on a single study and no alternative molecular mechanism capable of triggering LZ S phase entry has yet been established [35]. Resolving this question therefore remains an important priority. We consider it very unlikely that the reported findings simply reflect residual peptide‐MHCII expression on seemingly MHCII‐deleted cells because S‐phase entry was observed even among the rare MHCII‐deleted LZ cells persisting up to 6 days after tamoxifen treatment. Although awaiting experimental validation, our favored explanation is that GC B cells retain a residual reserve of prior “refueling” acquired during earlier rounds of T‐cell selection. In this model, refueling is replenished incrementally rather than fully reset from null after each DZ cycle and gradually decays over time, consistent with the progressive lengthening of G1 that permits AID reactivation. Consequently, LZ cells that initiate cyclic re‐entry in the absence of fresh T‐cell help would do so using a diminished residual reserve inherited from previous rounds of selection. Within the same framework, the presentation of particularly high levels of peptide–MHCII by some LZ cells may also promote their transient retention within the LZ, allowing more extensive refueling and thereby facilitating subsequent clonal burst behavior [31].

## 4 The Germinal Center as a Supporter of Antibody Diversity and an Expander of Functional Repertoires

The previous section discussed the long‐standing problem of how GCs select B cells according to the antigen‐binding strength of their BCRs ‐ the essence of affinity maturation ‐ a process that has historically been studied largely in the context of quasi‐monoclonal responses to model antigens. Because clonal diversity is generated during V(D)J recombination in the bone marrow, whereas affinity maturation in the GC simply acts on the naïve repertoire recruited into the response, the GC's contribution to shaping the diversity of the humoral response is easy to overlook. However, recent studies of physiological polyclonal responses have revealed that affinity maturation occurs in a far more clonally diverse environment than previously appreciated [6, 7, 8, 58].

Through multicolour fate‐mapping and photoconversion of individual GCs followed by single‐cell BCR sequencing, Tas and colleagues demonstrated that GCs responding to protein immunization are initially seeded by approximately 50–200 clonal lineages [8], rather just the handful of clones previously envisaged [79, 80, 81]. Although a substantial fraction of GCs became dominated by cells from a single clone through expansions arising from a single upstream selection event, or clonal burst, a large proportion of GCs avoided homogenizing selection. Instead, despite the inevitable reduction in clonal diversity imposed by repeated rounds of selection, many GCs retained substantial clonal diversity even at the peak of responses, including the persistence of clones with markedly lower‐affinity BCRs alongside higher‐affinity competitors. The persistence of cells bearing low‐affinity BCRs throughout the GC response was further supported by Kuraoka and colleagues [7]. Using single‐B cell cultures to screen on a high throughput scale BCRs from GC B cells responding to protein antigen in adjuvant, they reported a range of BCR avidities in the GC spanning more than three orders of magnitude. Although the overall avidity of the GC response increased over time, consistent with ongoing affinity maturation, this was not accompanied by the elimination of all weak binding clones. Instead, individual GCs continued to harbor B cells with widely differing apparent affinities (> 1000‐fold). Much of this spread was the result of interclonal variation, with members of individual clonal lineages varying by a smaller yet still significant range (10‐ to 30‐fold).

A snapshot frozen in time of the evolution process occurring in the GC is not straightforward to interpret. Cells present in the GC at a given point in time could be either on their way up (about to overtake the entire response) or on their way down (about to disappear from the response) [62]. Nonetheless, the wide range of affinities that can be observed between clones in the GC is unlikely to represent the product of a few days of maturation. Additionally, longitudinal studies have shown that naive B cells are readily recruited into established GCs at late time points despite expressing BCRs with very weak or undetectable affinity for the antigen [10, 11]. GCs therefore seem to have evolved not simply to make highly potent monoclonal responses: the retention of apparently lineages expressing BCRs that would, as antibodies, bind antigen only weakly appears to be a feature of GC biology.

More than simply preserving clonal diversity, GCs could even be argued to have a function in expanding diversity. To some extent, because affinity maturation can improve antibodies otherwise very weakly binding, the GC effectively expands the set of functional specificities that VDJ recombination produces. Indeed, the naïve repertoire contains BCRs which display extremely weak reactivity toward particular pathogen epitopes [9, 23, 82, 83]. These BCRs effectively correspond to “holes” in the naïve repertoire, since they would be useless if expressed as antibodies given that they cannot detectably bind their target when in solution; however, they often meet the binding requirements to drive a naïve B cell into a response thanks to the exquisite sensitivity of membrane‐bound BCRs during B cell activation [84]. Such antibodies can be tremendously useful once matured. For instance, HIV broadly neutralising antibodies (bNAbs) of the VRC01 class target the CD4 binding site of Env, yet fail to display measurable Env binding when somatic mutations acqured during affinity maturation are reverted to the germline sequence [85, 86]. The recovery of these antibodies in infected individuals demonstrates that this maturation pathway can be successfully traversed in vivo. These observations suggest that the capacity of GCs to retain and improve initially weak B cell clones, even in the presence of stronger competitors, may substantially broaden the functional antibody repertoire. From this perspective, one of the principal evolutionary advantages of the GC reaction may be not simply to maximize the affinity of already effective antibodies, but to rescue and refine otherwise inaccessible antigen specificities and enable alternative modes of antigen recognition.

An intuitive view of the GC as an environment of stringent competition for antigen gathering raises an obvious paradox: if higher‐affinity clones compete more effectively for limiting antigen, how do initially weak binders survive long enough to undergo affinity maturation? We return to this topic later in the Review.

## 5 Germinal Center Plasma Cell Differentiation

As mentioned above, GC B cells do not directly contribute to humoral immunity through appreciable secretion of soluble antibodies. Instead, they generate diverse pools of candidate antibody specificities and affinities from which the step of plasma cell differentiation ultimately selects to establish the serum antibody response. As such, an important question is the extent to which this process preserves or narrows the antibody affinity diversity generated within GCs, and the principles that determine which cells are ultimately selected.

### 5.1 Classic Studies of the Role of B Cell Receptor Affinity in Plasma Cell Differentiation

The rules governing GC plasma cell differentiation have long been of interest. Seminal studies from the Tarlinton and Brink laboratories in the 1990s and 2000s provided a careful analysis of how BCR affinity to the immunizing antigen determines the GC to plasma cell differentiation step in the context of clonally‐restricted responses either to the hapten NP (where B cells have highly restricted BCR gene segment usage) or by SW_HEL_ HEL‐specific B cells responding to a low affinity protein variant known as HEL^3x^ (where B cells have identical BCR genes) [16, 17]. The quasi‐monoclonal nature of these responses allows affinity maturation to be tracked with high resolution and in a quantitative manner, which are critical strengths. In anti‐NP responses, early affinity maturation steps are primarily driven by a tryptophan‐to‐leucine substitution at position 33 of the antibody heavy chain variable domain that confers an approximate 10‐fold increase in antigen‐binding affinity (1 μM to 100 nM), while SW_HEL_ B cells achieve a ~100‐fold (~100 nM to ~1 nM) improvement through a single tyrosine‐to‐aspartate change at position 53 [87].

In both settings, plasma cell populations accumulated the stereotypical affinity‐enhancing mutations earlier after immunization than the GC B cells, strongly arguing that BCR affinity plays a very important role in determining whether GC B cells adopt this fate [16, 17]. Moreover, within the same spleens, SW_HEL_ plasma cells were almost uniformly high affinity (> 80%) at a time point when most GC B cells (> 75%) had not yet acquired the Y53D mutation [16]. A logical inference from these results is that the selection strength required for plasma cell differentiation, a relatively infrequent event, is such that it is only achieved by GC B cells with the very highest BCR affinities. This model is evolutionarily intuitive because plasma cell niches are often said to have limited capacity and, by definition, high‐affinity antibodies will bind more of their target epitope at a given concentration. Subsequent studies, including those from our own group, have reported consistent findings under comparable experimental conditions [9, 88, 89].

### 5.2 Plasma Cell Differentiation During Responses to Pathogen‐Derived Antigens

While hapten and BCR knock‐in approaches provide the experimental resolution needed to dissect how BCR affinity influences selection outcomes, they do not reveal how these principles operate in physiological polyclonal responses elicited by vaccination or infection. A central inquiry, therefore, is how plasma cell differentiation balances recruitment of the highest‐affinity GC B cells with preservation of the affinity and clonal diversity generated within GCs. The distinction becomes especially important for complex proteinaceous antigens that present multiple epitopes that can be recognized by antibodies using diverse binding footprints and orientations. In such settings, the implications of a simple “high‐affinity plasma cell selection” model are far from obvious because maximizing antibody affinity might not maximize protective immunity. Instead, the rules governing plasma cell differentiation are expected to profoundly influence the breadth, epitope specificity and overall quality of the humoral immune response. Resolving this question therefore has important implications for rational vaccine design, particularly for strategies that seek to promote specific maturation pathways or the expansion of defined B cell clonotypes against complex antigens [85, 90]. Prompted by these questions, we conducted a study aimed at acquiring a snapshot of the plasma cells leaving GCs during influenza A infection [9].

Our study employed S1pr2‐CreERT2 bacterial artificial chromosome (BAC) transgenic mice [15], generously provided by Taka Okada and Tomohiro Kurosaki, that also carried Rosa26‐LoxP‐stop‐LoxP‐tdTomato and Blimp1‐mVenus reporter genes to enable GC B cell fate‐mapping and plasma cell identification [9]. S1pr2 encodes the G‐protein coupled Sphingosine‐1‐phosphate receptor 2 (S1PR2), whose expression is largely restricted to GC B cell stages among the B lineage and plays critical roles in constraining GC B cell positioning and growth [91]. As such, this genetic approach allowed us to specifically, temporally and indelibly label GC B cells at desired time points in immune responses and then isolate them alongside the plasma cells that they produced in the short time window left after tamoxifen treatment. In most experiments, mice received tamoxifen on day 11 post‐influenza A virus infection and responses in draining mediastinal lymph nodes were assessed 3 days later, on day 14. This setup provided as close as possible to a comparison between newly differentiated plasma cells and contemporaneous GC B cells. Mice also received the S1PR1 inhibitor FTY720 for the final 2 days to prevent plasma cell egress from lymph nodes, essentially trapping them where they were made [92]. These experiments focused on GC B cells and plasma cells that expressed BCRs specific for hemagglutinin (HA), which was determined through FACS staining using fluorescent multimeric antigen probes. Importantly, although IgG plasma cells eventually lose membrane BCR expression, this occurs at later times post‐differentiation. Antibody variable genes were sequenced and recombinant single fragment Fabs with corresponding sequences were expressed to enable monovalent binding affinity measurements through surface plasmon resonance (SPR).

In line with earlier findings from Tas et al. and Kuraoka et al.'s protein in adjuvant immunization studies [7, 8], influenza virus infection‐induced GCs supported concurrent maturation of many different B cell clones expressing BCRs with binding affinities (K_Ds_) spanning multiple (two to three) orders of magnitude. More surprisingly, the antibodies expressed by the plasma cells emerging from them spanned very similar affinity ranges. This meant that GC B cells with low BCR affinities (micromolar‐range K_D_) gave rise to plasma cells even when other GC B cells from the same responses expressed high affinity BCRs (nanomolar‐range), arguing that high BCR affinity is neither necessary nor sufficient for GC B cells to differentiate into plasma cells in this setting. Newly generated plasma cells were less clonally diverse than their parental GC B cell populations, as expected given the infrequency of plasma cell differentiation, however there were no detectable biases in the GC B cell clones from which plasma cells derived, beyond those attributable to differences in clonal abundance. In fact, some of the most successful clones in terms of GC participation and plasma cell abundance had comparatively weak BCR/antibody affinities. At the same time, GC B cells that failed to bind antigen tetramers were markedly underrepresented within the plasma cell compartment, consistent with the possible existence of a minimal affinity threshold for productive plasma cell differentiation. Collectively, the findings argue that GCs generate plasma cells that approximately mirror their own clonality and, as such, the maturation process as a whole—at least among cells with BCR affinities above a low minimal threshold. Consistent with this, plasma cells developed from multiple phylogenetic levels within clonal maturation, demonstrating that their development tracked the maturation trajectory rather than depended on rare super‐selection events.

These unexpected findings prompt an important question: why would the immune system evolve a selection process that invests resources in generating plasma cells expressing comparatively low‐affinity antibodies when seemingly superior alternatives are available? To answer this, we must consider what determines how protective an antibody will be against infection, because protection, not affinity itself, is ultimately at the heart of the evolutionary pressure that shaped the mechanisms underpinning immune responses. The antibodies that provide the greatest protection are typically those that neutralize pathogens by targeting epitopes in ways that disrupt essential pathogen functions. Although affinity maturation generally enhances the potency of such neutralizing antibodies (at least up to a point), increasing affinity alone cannot confer neutralizing activity when antibodies recognize intrinsically non‐neutralizing epitopes. B cells cannot tell the neutralizing potential of their BCRs; therefore, the GC's best solution may be to affinity mature clonally diverse B cell populations so as to explore multiple antibody binding modes and to generate plasma cells from many or all of them. Any other approach would represent a major gamble that risks focusing serum antibodies against a small number of “easier” epitopes that may represent poor targets for protection, which pathogens could exploit.

## 6 How Antigen Sensing Context May Uncouple BCR Affinity From Selective Advantage

At face value, the results from our “plasma cell snapshot” experiments may appear to contradict the conclusions inferred from the original studies of plasma cell differentiation, where nearly all plasma cells were generated by a minority of GC B cells expressing high‐affinity BCRs [16, 17]. These differences do not reflect quirks of S1pr2‐CreERT2‐based approaches because, in the same study, we performed very similar SW_HEL_ experiments to those of Phan et al. but with inclusion of GC fate‐mapping alleles, observing consistent outcomes [9]. It is also unlikely that these discrepancies are confined to early stages of antiviral responses; for example, due to heightened inflammation, because we observed similar phenomena at later infection time points (3 weeks) and after recombinant HA protein inadjuvant immunization. Rather, we propose that the different experimental systems provide complementary insights: the more reductionist model systems provide better resolution and population quantification for revealing selection criteria, whereas studies of complex polyclonal responses inform about how such selection rules are implemented when additional layers of complexity and regulation are present; that is, in settings that more accurately reflect “real” responses. We therefore argue that the observation that GCs generate plasma cells whose antibodies span a wide range of affinities is intimately connected to the broader question of how GCs balance the selection of modest, incremental affinity gains within clonal lineages with the continued co‐maturation of other clones bearing substantially higher or lower BCR affinities [7, 8].

In light of these arguments, we must consider how selection between clones may differ from selection within them. The retention of low BCR affinity cells cannot be explained purely by imperfect discrimination by T cells per se because, although individual affinity measurements are likely to be noisy (e.g., when B cells encounter particularly large antigen fragments or immune complexes that lead them to transiently present more peptide‐MHCII than neighboring cells), the progressive enrichment of higher‐affinity variants within clonal maturation trajectories supports that the cumulative selective pressure imposed over repeated rounds of competition is robust [8, 9, 62]. Rather, gaining insight into this apparent paradox requires considering both how GC B cells sense antigen and how competitive selection unfolds within a spatially and temporally dynamic GC environment. In the following sections, we discuss principles that may make GC selection dependent on factors beyond BCR affinity alone, before turning our attention to mechanisms that may preserve clonal diversity and thereby contribute to the broad affinity distribution of GC‐derived plasma cells.

### 6.1 Affinity‐Based Selection in a Complex Antigenic Landscape

To begin, we will explore the complex nature of antigen sensing and BCR affinity discrimination in GCs, considering how these processes could substantially impact the precision with which T cells distinguish the affinities of BCRs expressed by GC B cells from different clonal lineages.

Our understanding of the affinity maturation process has largely developed through the lens of a biochemical evaluation of antibody affinity, as measured through techniques such as surface plasmon resonance (SPR), Bio‐layer interferometry (BLI), ELISAs, etc. These methods are highly appropriate for characterizing the outcome of GC responses because they accurately quantify the binding properties of the antibodies ultimately produced. However, they may be less well suited to understanding the competitive process occurring within GCs, at least across B cell clones. Complexities related to how antigen is acquired and thus presented by GC B cells in vivo likely exist and may lead to disconnects between BCR affinity, antigen extraction and presentation of peptides to helper T cells. Consequently, affinity‐based selection could generate outcomes that appear permissive when judged by antibody affinity alone, because B cells expressing substantially different intrinsic‐affinity BCRs may nevertheless acquire similar amounts of T‐cell help.

To follow this argument, we must consider the nature of the antigen encountered by GC B cells. Even if delivered in free soluble form (*e.g*., a recombinant protein with adjuvant), GC B cells will predominantly engage with antigens in immune complexes immobilized on FDC reticular surface membranes [28]. The precise structural organization of such immune complexes has not been fully defined and will probably vary according to the challenge type, immunological setting and time point; nevertheless, they can be assumed to typically contain multiple antigen molecules interconnected by antibodies of differing affinities and specificities, and to include covalent decoration by complement proteins. Immune complexes are likely to be particularly heterogeneous during infections and following immunization with larger particle‐type vaccines (e.g., nanoparticle‐based), owing to the persistence and incorporation of associated proteins, lipids, nucleic acids and partially degraded antigenic material [93]. Regardless, all immune complex types are tethered to FDC membranes through multivalent interactions involving complement receptors (CR1/2) and Fc receptors (FcγRIIb), with complement receptors playing a more dominant role [41, 94, 95]. Although the individual receptor–ligand interactions are of relatively weak affinity, their combined valency generates strong overall attachment to the FDC membrane [96, 97, 98, 99]. In this context, GC B cells must exert sufficient mechanical force to disrupt the weakest interaction within the immune complex network, either detaching the antigen from the antibody, the complex from FDC receptors, or by pinching away a local region of antigen‐bearing FDC membrane, if they are to successfully acquire antigen for presentation to T cells. B cells generate pulling forces through Myosin IIa motor proteins acting on the actin cytoskeleton and transmitting tension through antigen‐bound BCRs [100]. It is not known how frequently contact leads to productive extraction, how many antigen molecules are typically acquired, whether higher‐order structures remain intact during this process, or what other (if any) non‐antigen molecules are typically dragged along. However, we can infer from measurements of BATF induction, which is T cell help dependent, that at least 5%–10% of LZ cells present at a given time have successfully engaged cognate T cell help [35]. Even when transient BCR–antigen interactions fail to trigger extraction, they may prolong LZ cell survival, assuming that BCR signaling known to occur at this time point is antigen‐dependent rather than tonic [68]. This basal signaling threshold could act as an affinity filter, purging cells with extremely low‐affinity BCRs or those targeting epitopes that are no longer available, from the selection pool.

Because FDC‐associated immune complexes are extensively tethered, antigen acquisition likely requires multivalent BCR engagement to generate sufficient pulling force while ensuring that individual BCR–antigen bonds remain intact under the forces required to disrupt the immune‐complex network [100]. By distributing the applied force across multiple receptor–ligand interactions, multivalent engagement reduces the force borne by any individual bond and thereby lowers the probability of rupture [98]. The resulting increase in overall binding strength, avidity, can be substantial. The power of this mechanism is illustrated by classic studies showing that B cells expressing BCRs with nanomolar affinities (strong but well within the physiological range) can generate sufficient force, when engaging highly multivalent antigen, to disrupt biotin‐streptavidin interactions [57, 96]. These interactions are among the strongest non‐covalent bonds in nature and exhibit dissociation constants approximately five orders of magnitude lower than those of the BCR–antigen interactions responsible for their disruption.

**FIGURE 2:**
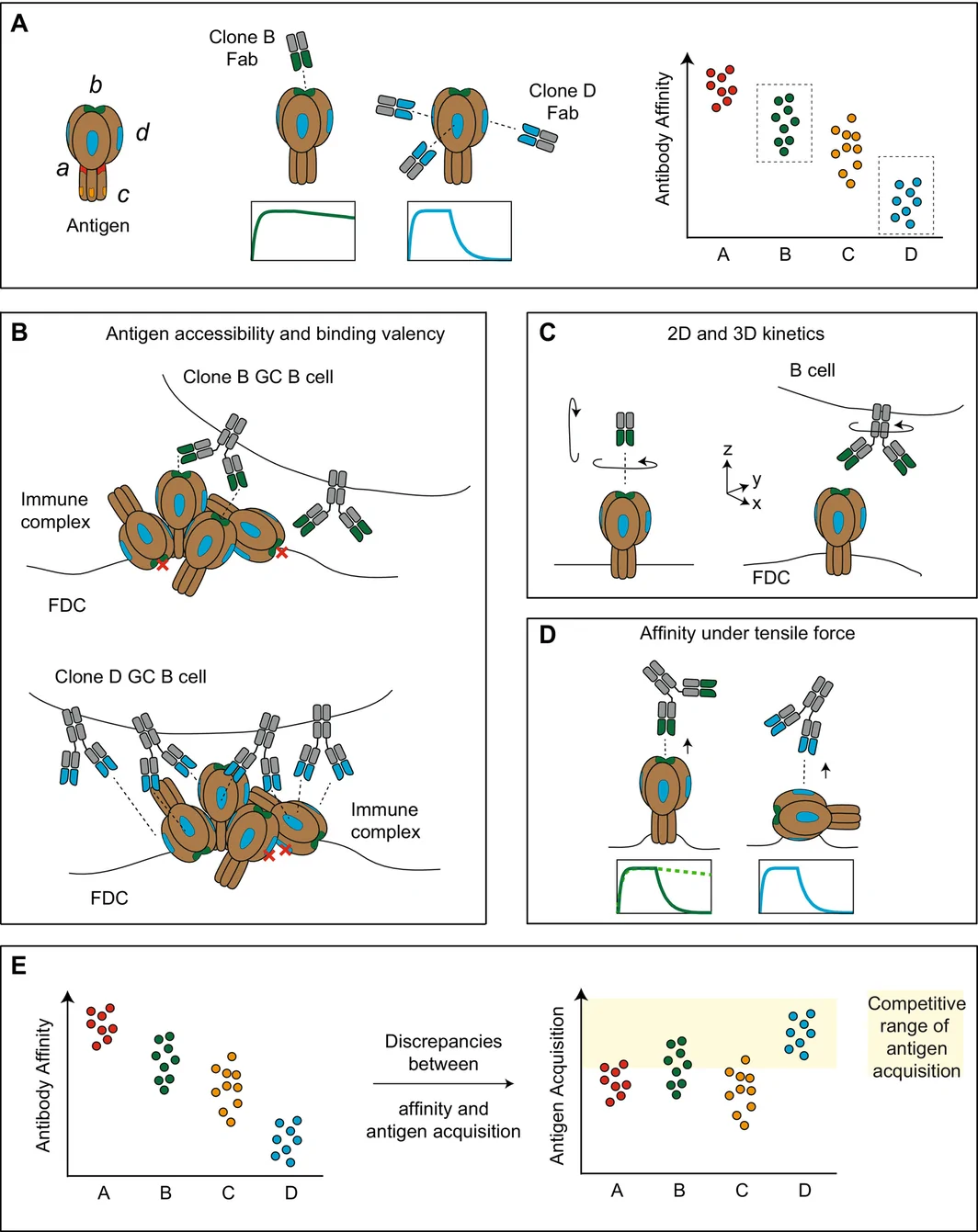
Discrepancies between antibody affinity and antigen acquisition potential of BCRs. (A) Consider a hypothetical antigen (left) with various different epitopes (a, b, c, and d) that may represent targets of antibodies developing in the GC. Affinity measurements using the antigen recombinantly expressed and soluble Fabs in the context of biochemical assays such as SPR or BLI (center) provides a good measure of antibody binding strength. B cells from various clones present in a GC can thus be ranked in order of the intrinsic affinity of the antibody they encode (right). This typically shows a picture where large differences exist between cells competing against each other. The dotted‐line boxes highlight two clones that we use to explain the phenomena that may lead to discrepancies between affinity and antigen acquisition. (B) During selection, GC B cell BCRs encounter and must extract immune complexes arrayed at the surface of FDCs. A clone D GC B cell, despite expressing an antibody of weaker affinity, gains a compensating valency advantage over a clone B cell. (C) In solution, Fabs or antibodies bind their antigen according to 3D kinetics in which docking requires rotational and translational diffusion to align the paratope and epitope. In contrast, the membrane of the B cell restricts the rotational movements of the BCR to a 2D plane. Under these kinetic restrictions, for fast‐kinetic antibodies, the soluble affinity measurements obtained via SPR or BLI may not be accurate predictors of the binding occurring in 2D. (D) In the LZ, B cells acquire antigen by applying tensile force on the BCR‐antigen bonds; this is not the case in conventional affinity measurement assays. In this case, clone B is depicted to be less stable under force than clone D. (E) Once the factors, other than affinity, that affect the efficiency with which a BCR can extract antigen from the membrane of an FDC are taken into account, the ordering of the clones that are competing against each other (now expressed as antigen acquisition) may be re‐shuffled. The cells able to compete in the GC would be the top cells in this new distribution (notably, clone D, despite its weak affinity, performs better in the competition for T cell help due to its advantages in the other parameters described).

This has important implications for how BCR affinity and antigen acquisition can decouple across clonal lineages, because it implies that antigen extraction efficiency depends not only on BCR affinity but also the level of valency achieved with an immune complex, a variable that is likely to differ across B cell clones with identical BCR affinities. A first simple source of variation arises from epitope architecture itself (Figure 2A,B). While some epitopes will be present and available for binding on each protomer of a multimeric antigen, others will not. For instance, the CD4‐binding site recognized by VRC01‐class anti‐HIV antibodies is present on each of the three Env protomers, allowing simultaneous occupancy of all three sites [101], whereas the quaternary apex epitope recognized by PG9/PG16‐like antibodies spans multiple protomers and therefore permits only a single binding event per trimer [102, 103]. Similar steric constraints and varying permitted angles of approach apply to influenza haemagglutinin [104] and nanoparticle‐displayed antigens [105], influencing how effectively avidity can amplify extraction forces independently of intrinsic affinity.

This structural complexity is further compounded by the temporal dynamics of the antigen, which may alter epitope availability over time. For example, certain “cryptic” B cell epitopes might only be transiently or periodically accessible during conformational “breathing” unless stabilized by antibodies, while others may require partial protein denaturation or degradation to expose buried residues [106, 107, 108, 109]. Although B cell follicles maintain unusually low extracellular protease activity to favor preservation of native antigen structure, some degradation resulting from enzymatic or mechanical insult is inevitable, causing B cells to affinity mature toward degradation products—again contributing to variations in functional avidity between clones [110, 111, 112]. While clones targeting degradation‐dependent epitopes are likely to escape detection via antigen multimer FACS staining, they may contribute to the appearance that GCs sustain B cells with negligible BCR binding affinity [7, 12]. Though it is not intuitive why targeting epitopes that may only be present on a fraction of all antigen molecules would benefit B cells early on, such cells may preferentially gain advantage at later response stages when antibodies begin masking initially dominant epitopes [11], which we discuss in more detail later. Consequently, clones whose BCRs target cryptic or temporal epitopes may experience a progressive increase in relative antigen access, allowing them to compete disproportionately for Tfh help during later response stages [10, 11].

Even when epitopes are similarly available and allow complete occupancy by soluble Fab fragments in vitro, equivalent engagement by membrane‐bound BCRs in vivo cannot be assumed. Membrane‐bound BCRs are substantially bulkier than simple Fab fragments and engage antigen within the geometrically constrained environment of the immune synapse, where receptor orientation, intermembrane spacing, and epitope accessibility all influence binding. Consequently, effective antigen recognition depends not on the intrinsic three‐dimensional affinity of the interaction but rather its effective two‐dimensional affinity in the membrane context [113] (Figure 2C). In solution, association rates are determined largely by translational and rotational diffusion of the interacting molecules. By contrast, at the immune synapse, receptor and ligand are confined to opposing surfaces, making encounter rates and binding probabilities highly dependent on molecular orientation, spatial organization, and membrane geometry. These constraints are likely to become particularly important when multiple BCRs must engage simultaneously to maintain attachment to mechanically resistant immune complexes. Consequently, factors such as epitope spacing, angle of approach, local membrane topology, and the architecture of antigen and the BCR itself may substantially influence the effective binding kinetics experienced by different B cell clones, independently of intrinsic affinity. Additional external factors such as covalent attachment of complement fragments, as well as weaker associations with other serum proteins and antibodies (expanded on later) [114], could further impact steric accessibility and/or structural dynamics. Moreover, in some cases antibody binding may dynamically influence antigen structure itself by stabilizing particular conformational states, exposing cryptic epitopes, or reducing accessibility of neighboring epitopes, thereby altering the antigenic landscape perceived by subsequently arriving B cells [106, 107, 108, 109]. Occasionally, this two‐dimension association principle may itself become a substrate for affinity maturation; for example, B cells responding to the highly repetitive circumsporozoite protein of *Plasmodium falciparum* acquire mutations that optimize the geometry of BCR engagement such as to promote more effective clustering and multivalent binding [115]. Consequently, two epitopes that appear similarly accessible to soluble Fabs in biochemical and structural assays could differ substantially in the degree of multivalent engagement achievable by B cells in vivo.

Finally, the required application of force during antigen extraction further complicates the relationship between intrinsic affinity and productive antigen acquisition [96, 100]. Receptor–ligand dissociations are generally accelerated by tensile force, a phenomenon known as slip‐bond behavior; however, the magnitude of this effect may vary among interactions [116] (Figure 2D). Consequently, two B cells recognizing the same antigen using BCRs that provide similar *K*_D_ measurements in solution could differ in their ability to maintain productive attachment during extraction.

Collectively, these examples illustrate why we should not assume a linear relationship always exists between BCR affinity and antigen extraction capacity in vivo. While affinity maturation generally improves the competitive capacity of individual clones, differences in epitope geometry and accessibility mean that B cells may also achieve competitive success through mechanisms other than increased intrinsic affinity (Figure 2E). Analogous to other ecological systems competing for limiting resources, affinity may represent only one of several traits contributing to competitive fitness. The relative importance of these alternative parameters, and the extent to which they can compensate for lower intrinsic affinity, remains to be determined.

### 6.2 Clonal Success Is Rebalanced Through Antibody Feedback

This discussion has so far focussed on mechanisms that may lead GC responses to systematically favor certain clones over others due to features that complement antibody affinity in determining acquisition and the subsequent receipt of T cell help. We now turn to complementary mechanisms enacted by the immune system to preserve diverse trajectories of clonal affinity maturation despite competitive pressures.

**FIGURE 3:**
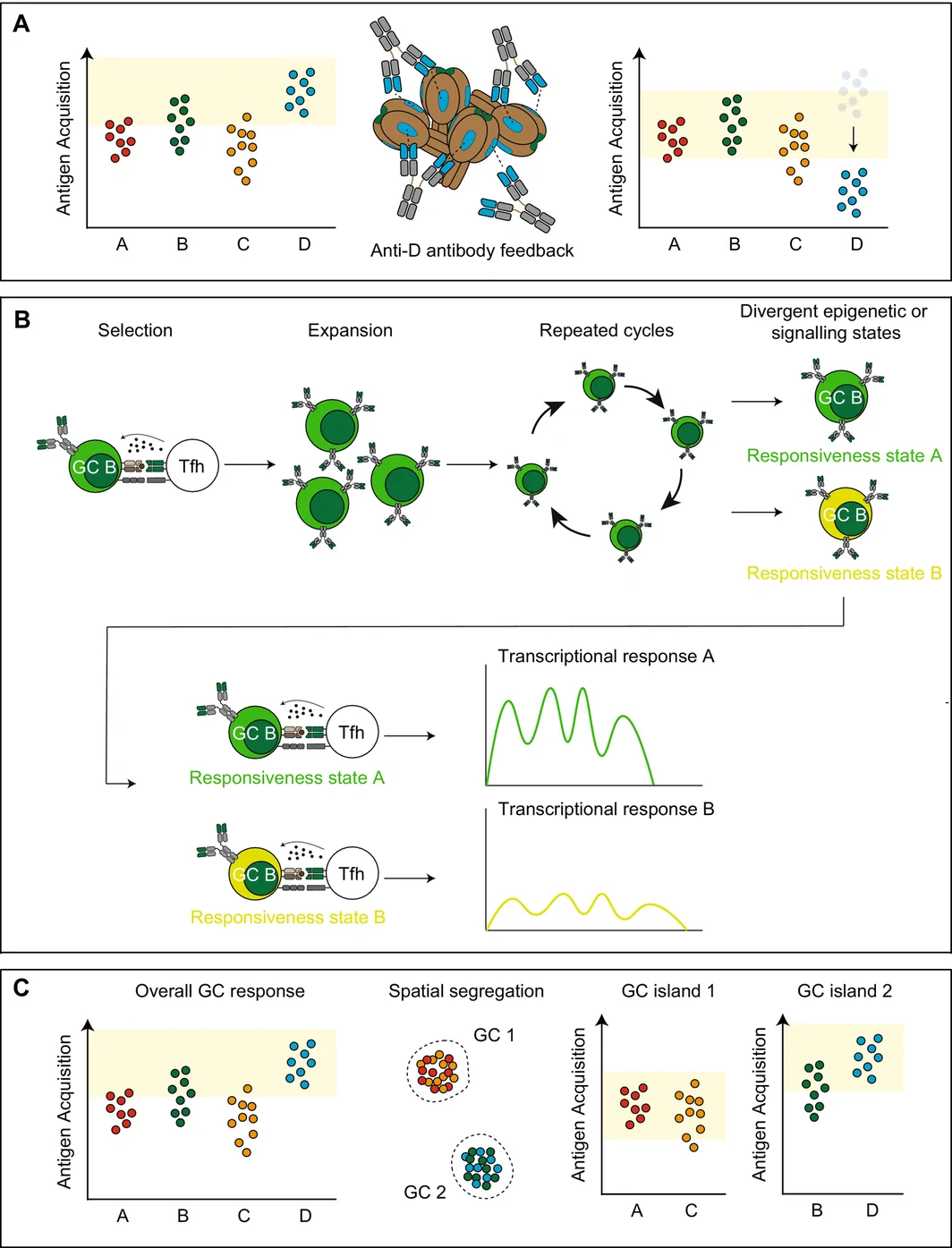
Mechanisms promoting clonal diversity in the GC reaction. (A) Consider the distribution of GC B cells, ordered by antigen acquisition efficiency, established in Figure 2E. In this scenario, the most successful clone, D, would dominate the response, produce most plasma cells, and therefore generate the most constraining antibody feedback. Because antibodies from this clone do not mask the epitopes of the other clones in the response, the antigen acquisition capacity of clone D is reduced relative to its competitor clones, allowing them to become competitive in the following selection events. N.B., Antibody affinity as well as abundance determines whether antibodies confer negative feedback, which for simplicity is not indicated here. (B) Different GC B cells in the response will have different histories of selection events and have undergone different amounts of proliferative cycles. This could be recorded as epigenetic changes (*e.g.,* due to accumulation of IRF4 upon BCR signaling and receipt of T cell help) that lead different LZ cells to respond differently to identical T cell help signals. These cells might thus respond differently to the same selection cues. (C) The spatial segregation of GCs likely contributes to the retention of clonal diversity in the response overall. In this example, the distribution of GC B cells from Figure 2E is broken down into two individual GC islands where the antigen acquisition capacity required to be competitive differ. As a result, clones A and C are allowed to successfully mature.

Clonal success in GCs is accompanied by plasma cell differentiation, which itself alters the competitive landscape of the GC as a result of the arising antibodies preferentially occluding their epitopes (or close‐by epitopes) on FDC‐associated immune complexes, a phenomenon known as antibody feedback [117, 118, 119, 120, 121]. Feedback initially derives from pre‐existing serum antibodies present from earlier exposures [122, 123, 124, 125, 126, 127]; however, this is subsequently supplemented by reactivated memory B cells giving rise to plasmablasts [128], and eventually from ongoing GC‐derived plasma cells [124, 129, 130, 131, 132]. The outcomes of antibody feedback vary according to both antibody concentration and affinity, with lower‐affinity or less abundant antibodies often enhancing GC responses by promoting immune‐complex formation and deposition (at least if present prior to challenge), whereas higher‐affinity and/or more abundant antibodies generally suppress responses of the same specificity [94, 124, 125]. Negative feedback is therefore expected to preferentially impact clones or epitope specificities that have been particularly successful thus far or whose antibodies have strong effective binding affinities for the antigen, and thereby favor B cells targeting distinct epitopes and those with a BCR affinity disadvantage (Figure 3A).

At first glance, this might be expected to progressively restrict antigen availability within established GC, making it difficult to envisage how GC B cells could establish the multivalent BCR engagement required for antigen extraction. However, GC B cells clearly continue to extract sufficient antigen to sustain affinity maturation, implying that complete epitope occlusion is rarely achieved. One plausible explanation is that steric and geometric constraints limit the simultaneous occupancy of neighboring epitopes, particularly when antibodies bind bivalently across adjacent antigen molecules or subunits. Consequently, a proportion of epitopes may be occupied only through monovalent antibody interactions, resulting in substantially faster dissociation and epitope re‐exposure that would preferentially benefit GC B cells that are in direct competition with lower affinity antibody or those targeting initially subdominant epitopes. Whether these opportunities are successfully exploited would then depend not only on intrinsic BCR affinity but also on the effective two‐dimensional association kinetics of competing GC B cells that are trying to dock onto it, as discussed earlier. Consistent with this, access to refueling appears to be co‐limited by antigen availability and T‐cell availability, as evidenced by the increased frequency of B cells receiving T‐cell help when excess antigen is provided [35].

Recent studies have provided compelling evidence that antibody feedback dynamically regulates GC immunodominance. Barbulescu et al. developed a novel mouse model where practically all Blimp1‐expressing plasma cells (or, depending on the experimental configuration, those derived from GCs or specific BCR knock‐in clones) undergo apoptosis during or very soon after differentiation, meaning that they don't contribute meaningful amounts of serum antibody [129]. Although their experiments did not find antibody feedback directly altering rates at which affinity‐enhancing mutations accumulate within individual clones (which appears to occur in some settings [124, 130]), it had a profound impact on clonal representation within GCs. Preventing plasma cell differentiation approximately doubled GC participation of engineered B cells expressing BCRs specific for a single HA epitope, without measurably affecting GC B cells targeting a structurally distinct epitope. Along similar lines, Yu et al. used a panel of B cells expressing pre‐rearranged BCRs spanning different affinities and epitope specificities for an HIV antigen and found that high‐affinity B cells suppressed lower‐affinity competitors only when they recognized the same epitope [124]. An equivalent pattern was reproduced by administering matched monoclonal antibodies before immunization, supporting antibody‐mediated feedback as the underlying mechanism. Collectively, these findings indicate that antibody‐mediated negative feedback acts through epitope‐restricted competition, limiting the expansion of dominant clones and thereby enhancing opportunities for recruitment and persistence of alternative specificities. Consequently, even partial (~10–30‐fold) reductions in antibody‐mediated feedback were sufficient to reduce GC clonal diversity [131].

### 6.3 Additional Mechanistic Hypotheses for Permissive Selection and Clonal Diversity

It has been suggested that the process of selection itself may be implemented in a manner that is not excessively stringent or restrictive; that is, that it is somewhat permissive [4, 6, 35, 36, 133]. In light of the preceding discussion, we view permissive selection not as an explanation for the broad affinity distributions observed within GCs—although this remains to be established experimentally—but rather as a mechanism to accommodate residual differences in antigen acquisition and presentation that may persist between competing clones. In essence, it may provide a degree of tolerance to inevitable competitive differences. We argue that separating cyclic re‐entry from GC B cell refueling may contribute by ensuring that B cells are not immediately eliminated simply because they fail to rank among the strongest competitors during a single LZ cycle [35]. Instead, they retain the opportunity to acquire antigen in subsequent cycles, allowing transient competitive disadvantages to potentially be overcome. The benefits of this may be particularly apparent when proliferation and SHM are intrinsically coupled (outside of proliferative bursts) [44, 45], because the advantage of more rapid proliferation is partially offset by the increased opportunities for deleterious mutation and, consequently, negative selection.

Given the lack of direct experimental evidence defining the extent to which the mechanisms discussed above account for the clonal diversity observed in GCs, it is worth considering additional factors that might contribute to selection permissiveness. These ideas are necessarily speculative and should be viewed as hypotheses rather than established features of GC biology. In principle, permissiveness could arise if individual GC B cells or clonal lineages adapt their signaling pathways, epigenetic state, or the manner in which they respond to selection cues as a consequence of previous selection or exposure history (Figure 3B). A particularly striking example of apparent permissiveness comes from wildtype:*Aicda* ^*−/−*^ mixed bone marrow chimeric mice, in which AID‐deficient GC B cells remain surprisingly competitive for several weeks despite being incapable of SHM [134, 135]. This has been proposed to reflect reduced apoptosis [136], suggesting that defects in BCR affinity can, at least partially, be offset through changes in cellular behavior. We have already discussed that GC B cells undergoing clonal bursts transiently suppress AID activity during periods of rapid DZ expansion to safeguard selected antibody genes while preserving their proliferative advantage [44, 45]. Whether GC B cells similarly modulate SHM rates in other settings, or in ways that differ between clones, remains unknown. More broadly, sequence‐dependent differences in mutational susceptibility, tolerance of amino acid substitutions, or sensitivity to changes in codon usage and tRNA availability could all, in principle, influence clonal fitness independently of intrinsic BCR affinity [137].

A possible route through which adaptive changes could arise is progressive epigenetic remodeling, such as occurs in other settings to enable individual cells to alter their competence to respond to subsequent signals [138]. Consistent with this idea, GC B cells were recently reported to become progressively poised for Prdm1 (Blimp1) expression and plasma cell differentiation over time as a result of a gradual shift in the Blimp1–Bach2 regulatory balance [139]. This transition is driven by Irf4, whose expression is induced downstream of BCR engagement and T‐cell help and which acts as a pioneer transcription factor to progressively increase chromatin accessibility at key plasma cell‐associated loci—essentially leading cells to accumulate an epigenetic record of their previous selection and proliferative history. AID has also been proposed to contribute to (or is associated with) epigenetic remodeling through CpG demethylation [140, 141], possibly through cooperation with the methylcytosine dioxygenase TET2 [142], thereby promoting the expression of genes associated with plasma cell differentiation. Likewise, studies of activated B cells outside the GC have shown that cell division history is accompanied by progressive CpG demethylation and chromatin remodeling that precede and facilitate plasma cell differentiation [143, 144]. Therefore, while there is currently no evidence of such epigenetic modifications directly influencing clonal competition within GCs, these studies suggest that previous cellular experiences can become molecularly encoded within B cells and subsequently shape their future responses to selection and differentiation cues.

### 6.4 Responses Split Across GC Islands

The structural organization of humoral immunity itself must contribute to maintaining clonal diversity and establishing diverse maturation trajectories, because affinity maturation is spatially compartmentalized across multiple GCs [91, 145]. Because each B cell follicle contains only a single GC, affinity‐maturing B cells compete directly only with neighboring cells within their local reaction rather than with B cells undergoing affinity maturation elsewhere (Figure 3C). This partitions the adaptive landscape into multiple semi‐independent evolutionary “islands” [8]. Reactive murine lymph nodes typically contain around 10–20 GCs, mouse spleens contain dozens, and human lymphoid tissues may contain hundreds, providing considerable scope for parallel evolutionary trajectories [146, 147]. This spatial segregation may have evolved in part to confine mutating B cells within the specialized GC niche, consistent with the long‐recognized inability of GC B cells to survive for extended periods outside the follicle, to which S1PR2‐mediated confinement likely contributes [91, 148]; however, it probably serves an important function in the clonal dynamics of antibody responses by restricting the extent to which a single successful clone can dominate the overall response, while still permitting local clonal dominance within GC foci. This organization may represent an effective compromise, rewarding affinity improvements without excessively restricting clonal diversity. Consistent with this idea, B cells bearing identical BCR sequences often differ markedly in the degree to which they dominate separate GCs [8, 62], demonstrating that members of the same clone can follow distinct evolutionary trajectories when distributed across multiple competitive environments. That does not, however, mean that each GC evolves in complete isolation; B cells undergo clonal expansion before GC seeding, allowing members of the same clone to seed multiple GCs [8], while antibody‐mediated feedback and [124, 129, 130, 131], potentially, migration of Tfh cells between neighboring GCs are likely to permit some communication between reactions [145]. Nevertheless, newly generated somatic variants compete for T‐cell help only within their local GC.

Combining a two‐photon‐mediated photoactivatable GFP (PAGFP) approach, previously pioneered by Victora et al. to label individual GCs [31], with GC fate mapping, we assessed plasma cell generation from single GCs following influenza A infection [9]. Because plasma cells leave GCs during or shortly after differentiation, we used BCR clonality and shared somatic mutation patterns to identify plasma cells derived from individual GCs. Although technical challenges limited the depth of this analysis, the results nevertheless provide evidence that individual GCs give rise to plasma cells of comparatively low BCR affinity, even when the same GC contains other B cells with BCR affinities many‐fold higher. Therefore, spatial segregation should probably be seen as a contributor to the maintenance of clonal and affinity diversity rather than the primary determinant. It may also provide an opportunity for antibody feedback to play a more nuanced, temporally localized role if newly differentiated plasma cells begin secreting antibody before leaving the GC [124]. Ultimately, spatial compartmentalisation provides the framework within which the various determinants of GC selection discussed herein, including antigen presentation dynamics, antibody feedback, permissive selection and local competition, interact to shape the distinct clonal trajectories of individual GCs.

## 7 Reconciling Findings From Model and Complex Response Experimental Settings

Taking these various discussions into account, we return to the question of why BCR affinity has such contrasting importance in determining the plasma cells generated in GCs following influenza A infection or HA protein in adjuvant immunization compared with quasi‐monoclonal systems and speculate on what this means more broadly for GC plasma cell differentiation. Despite the simplified nature of the SW_HEL_ and NP systems, we see no reason to believe that they operate under fundamentally different selection rules from more complex immune responses. We therefore propose that B cell clones expressing comparatively low‐affinity BCRs generate plasma cells not because they escape affinity‐based selection, but because they successfully compete within it [9]. Under this view, the key distinction between these settings is not whether affinity‐based selection occurs, but the extent to which differences in BCR affinity are translated into competitive advantage.

Our findings also raise a second question. Even within individual clones, plasma cell differentiation was not restricted to the very most matured stages, suggesting that affinity alone cannot explain intraclonal plasma cell output. This may in part reflect an inherent limitation of the experimental system in that fate‐mapping was performed across 3 days; however, this is probably not the whole explanation. Rather, we suspect that the SW_HEL_ system magnifies the relationship between BCR affinity and plasma cell differentiation. SW_HEL_ responses undergo exceptionally rapid and uniform affinity maturation. In our hands, HEL^3x^‐binding SW_HEL_ GC B cells, reflecting acquisition of the affinity‐conferring Y53D mutation, were readily detected by day 7 after immunization and increased from approximately 10% to 75% of the GC population between days 7 and 9 [9]. This provides remarkable experimental resolution, but it is unlikely to be representative of the more gradual maturation trajectories followed by many physiological clonal lineages.

Several features of the SW_HEL_ system may contribute to this particularly efficient affinity maturation. First, an approximately 100‐fold increase in antigen affinity can be achieved through a single nucleotide substitution, providing a highly accessible evolutionary path toward the high‐affinity state [87]. Second, adoptive cell transfer results in extensive seeding of individual GCs by SW_HEL_ B cells, increasing the probability that advantageous mutations arise early and expand. Finally, the physical organization of (monomeric) HEL^3x^ on the surface of sheep erythrocytes may favor particularly efficient affinity discrimination during antigen extraction. Although the antigen density is not well characterized, its presentation on a deformable membrane could substantially influence antigen extraction by affecting BCR clustering, force generation and antibody‐mediated epitope masking. Furthermore, because each antigen molecule (an erythrocyte or erythrocyte fragment) yields numerous peptide–MHCII complexes following processing, even a single successful antigen acquisition event may provide a substantial increase in antigen presentation. Similar considerations may apply to anti‐NP responses, where the immunizing antigens likewise display a high valency of NP epitopes. Collectively, these features are likely to steepen the relationship between BCR affinity and competitive success, thereby strengthening the association between affinity maturation and plasma cell differentiation observed in these models.

We propose that these collective observations may reconcile the apparently discrepant conclusions drawn from reductionist and physiological experimental systems. Plasma cell differentiation is influenced by BCR affinity, but affinity is interpreted within a competitive context that extends beyond biochemical binding strength alone. Antigen organization, immune complex architecture, epitope geometry, antibody feedback, and the composition of competing clones all have the potential to modify how efficiently GC B cells acquire antigen and compete for T‐cell help. This is unlikely to lead to perfectly matched levels of antigen presentation by B cells from different clones; however, small differences may be tolerated under a framework of refueling‐based selection that does not include a binary re‐entry bottleneck [35]. Whether the biophysical factors discussed herein are sufficient to fully explain the broad range of plasma cell affinities observed in physiological responses, or whether the GC selection process also incorporates permissive adaptations to how cells respond to selection, such as those discussed above and in other reviews [6], remains unresolved. Ultimately, distinguishing between these possibilities will require either direct evidence that co‐residing GC B cells with different intrinsic affinities acquire (or not) antigen with comparable efficiency in vivo, or direct demonstration that the selection process itself does (or not) incorporate permissive mechanisms that influence competitive outcomes.

Two other recent studies also revisited the relationship between BCR affinity and plasma cell differentiation within GCs. The first arrived at conclusions broadly consistent with our own [149]. Using NP–OVA immunization, the authors examined antibody affinities within putative plasma cell precursor populations, reasoning that these cells provide a more direct readout of GC exit than mature plasma cells. Although high‐affinity BCRs were enriched relative to matched GC populations, substantial numbers of precursor cells exhibited low or even undetectable antigen binding. Notably, these findings align closely with the idea that plasma cell differentiation is affinity‐sensitive rather than affinity‐exclusive. Affinity contributed measurably to selection, yet lower affinity cells continued to enter the plasma cell pathway in substantial numbers. In the second study, Sutton et al., who also studied putative pre‐plasma cells in a system using a fixed BCR heavy chain paired with different light chains that confer varying affinities for a malaria antigen, found no evidence for enrichment of BCR affinity during the GC‐to‐pre‐plasma cell transition [150]. The absence of affinity selection in this setting may have several explanations. Most obviously, putative pre‐plasma cell populations must contain many cells that never complete differentiation, given their abundance and how rarely plasma cells differentiate. Consequently, enrichment observed at the precursor stage may underestimate the extent of selection ultimately imposed. Alternatively, BCR affinity may become a weaker discriminator once all competing cells exceed a relatively high affinity threshold, as was the case in the malaria‐specific system studied by Sutton et al. Finally, additional features of the immune context may influence the extent affinity contributes to plasma cell selection.

Finally, we noted the frequent recurrence of identical sequences among GC‐derived plasma cells, suggesting that further clonal expansion occurs at the plasma cell stage [9]. This conclusion was subsequently confirmed using 5‐ethynyl‐2′‐deoxyuridine (EdU) labeling and a genetic stochastic clonal lineage labeling approach. This finding was initially surprising because Blimp1 is well known to repress *Myc* expression in terminally differentiated plasma cells [151]. However, others have since reported similar observations [152], with Maclean et al. arguing that this process is initiated within GCs and preferentially expands plasma cells expressing high‐affinity antibodies [89]. It is possible or even likely that this influences the extent to which newly generated plasma cells contribute to the LLPC compartment in the bone marrow. Even so, when considering the interclonal competition occurring in polyclonal responses, we believe post‐GC expansion is unlikely to be the primary explanation for the success of low‐affinity clonal lineages in generating plasma cells because, first, some low‐affinity clones gave rise to some of the largest plasma cell expansions and, second, the same low‐affinity clones that generated the most plasma cells were also the most successful within the GC itself. Nevertheless, it will be important to determine whether LLPCs predominantly arise from cells that undergo substantial post‐GC expansion and to identify which antibody specificities persist within that compartment over the long‐term following a complex antigenic challenge such as those elicited by human vaccines.

## 8 Closing Remarks

In this Review, we have outlined how single‐cell antibody sequencing, genome editing and genetic fate‐mapping have paved the way for a better understanding of how clonality and affinity evolve during the GC reaction and into the memory and plasma cell compartments. Although these findings initially appeared difficult to reconcile, they have substantially improved our understanding of the true composition of protective humoral immunity and have prompted renewed consideration of the constraints under which GC B cells compete in vivo. In turn, this has generated new hypotheses for how antigen presentation, antibody feedback and vaccine design might be harnessed to promote desirable maturation trajectories and patterns of clonal dominance [82, 94, 111]. As we have emphasized throughout, further work will be required to determine which of these hypotheses are correct and to define their relative contributions to shaping GC output.

We have argued that preserving clonal diversity among plasma cells may represent an important evolutionary strategy, allowing the immune system to hedge against uncertainty regarding which antibody specificities will prove most protective. This concept, however, carries an important implication: such a strategy is likely to be most effective only if GCs produce plasma cells at sufficient frequency to capture a substantial fraction of the diversity generated during affinity maturation; differentiation events too rare would naturally fail to capture much clonal diversity. Using the S1pr2‐tdtomato fate‐mapping system, we were able to get a sense of the efficiency with which GCs in various settings were producing plasma cell populations, expressed as a ratio of recent plasma cells over successfully fate‐mapped GC B cells [9]. By this measure, we found influenza‐induced GCs to be much more plasma cell‐productive than GCs responding to immunization with protein in adjuvant. This applied to various other common vaccination strategies (unpublished), suggesting that it is likely to limit the efficacy of multiple vaccine candidates where desired clonal lineages which might not be the most immunodominant must make it to the plasma cell stage. With this limitation in mind, we see two main strategies one might employ to improve vaccine candidates in this regard. One is to improve the dominance of the desired clonal lineages; the second, to improve the output of plasma cells overall.

Given the importance of antigen context and presentation in determining GC selection, manipulating antigen form represents a promising avenue for improving the competitive success of desired clonal lineages. Antigen delivery strategies already provide opportunities to influence this process. For example, escalating‐dose immunization promotes more efficient antigen deposition on FDCs and better preservation of native antigen structure, thereby improving the focusing of GC responses toward relevant epitopes [153, 154]. Likewise, some nanoparticle‐based vaccines appear to benefit from efficient complement activation through optimal spatial organization of surface glycans, enhancing immune‐complex deposition within follicles [111, 155]. Our discussion also highlights additional, under‐explored opportunities. The way GC B cells acquire antigen depends not only on intrinsic BCR affinity but also on avidity, force‐dependent bond stability and the two‐dimensional geometry of receptor engagement [96, 113]. These considerations suggest that antigen architecture itself could be engineered to favor particular B cell specificities [105]. Ultimately, it will be important to determine what constitutes an optimal antigen array for B cell extraction, including the spacing, orientation and accessibility of epitopes, and whether these features can be engineered to favor desired clonal lineages while minimizing antibody‐mediated feedback. Moreover, a better understanding of the changes in form that vaccine candidates undergo at different stages of the response might instruct approaches to ensure the favoring of the desired lineages persists as long into the GC reaction as possible.

In light of the discussion above, we also proposed that control of plasma cell output might be extremely useful in vaccine design. The increase in plasma cell production we observed in influenza infection compared to protein immunization had no link to antibody affinity and appeared to reflect a higher frequency of differentiation rather than simply an improvement in post‐GC expansion [9]. It thus seems that GCs can modulate the propensity of B cells to differentiate, through signals defined by the nature of the challenge, although we currently have no mechanistic explanation for this effect. Elucidating the basis of this phenomenon could enable us to adjust the plasma cell output of vaccination systems. This could obviously help enable sufficient antibody titers to be reached such that sterilizing immunity is feasible in the case of vaccines where such a threshold is high, but it could also provide opportunities to generate vaccines that capture greater clonal diversity. However, to fully exploit this opportunity, we must also understand the consequences of increasing plasma cell output from GCs. One obvious consequence would be enhanced antibody‐mediated feedback, which may become undesirable in vaccine strategies requiring prolonged affinity maturation. For example, HIV vaccination approaches that seek to shepherd broadly neutralizing antibody (bNAb) lineages through complex sequential maturation pathways may benefit from limiting plasma cell differentiation until the desired maturation trajectory has been achieved, thereby minimizing premature antibody feedback [124].

More fundamentally, attempts to manipulate plasma cell output require a better understanding of how differentiation is coupled to positive selection. The prevailing view is that particularly strong LZ selection events directly induce plasma cell differentiation through downregulation of Bcl6 and induction of IRF4 [88]. However, such a model raises an obvious question: how can the GC generate plasma cells without simultaneously depleting its most competitive B cells? An alternative possibility is that plasma cell commitment occurs secondary to the subsequent DZ expansion phase, allowing a single strong selection event to increase both continued GC participation and plasma cell output [156]. Such a mechanism would be analogous to non‐GC B cell responses, in which plasma cell differentiation becomes progressively linked to cell division through division‐dependent transcriptional and epigenetic reprogramming [143, 144]. The recent description of mutation‐free proliferative bursts in the DZ makes this possibility particularly attractive [44, 45]. Accordingly, although not direct proof, DEC‐205‐mediated augmentation of T‐cell help results in simultaneous increases in GC participation and plasma cell production, rather than the latter occurring at the expense of the former [31]. Whether plasma cell differentiation competes directly with recycling or instead branches from subsequent DZ expansion therefore remains unresolved. Resolving this question will be critical for vaccine design, because strategies aimed at increasing plasma cell output may either enhance humoral immunity or prematurely curtail affinity maturation, depending on how these two processes are mechanistically linked.

## Funding

The Bannard Lab is supported by Wellcome Trust, 315850/Z/24/Z, Biotechnology and Biological Sciences Research Council, UKRI1929.

## Conflicts of Interest

The authors declare no conflicts of interest.

## Data Availability

Data sharing not applicable to this article as no datasets were generated or analyzed during the current study.
